# Understanding the Patient Perspective When Designing Future Rehabilitation Interventions after Hip or Knee Replacement Surgery—A Patient and Public Involvement Exercise

**DOI:** 10.3390/medicina59091653

**Published:** 2023-09-13

**Authors:** James P. Gavin, Louise C. Burgess, Tikki Immins, Thomas W. Wainwright

**Affiliations:** 1 School of Health Sciences, University of Southampton, Southampton SO17 1BJ, UK; 2 Department of Rehabilitation and Sport Sciences, Bournemouth University, Bournemouth BH12 5BB, UK; 3 Orthopaedic Research Institute, Bournemouth University, Bournemouth BH8 8EB, UK; 4 Physiotherapy Department, University Hospitals Dorset, Bournemouth BH7 7DW, UK

**Keywords:** PPI, patient involvement, joint replacement surgery, allied health professionals, recovery

## Abstract

*Background and Objectives*: Following discharge from hospital, there can be variability in the rehabilitation of patients who have undergone total hip or knee replacement surgery. We invited patients who had had hip or knee replacement surgery to take part in patient and public involvement sessions to help us understand their recovery needs and how rehabilitation services could potentially be improved to meet these needs better. *Materials*: Patients (*n* = 14) were invited to one of two patient advisory group sessions which took place in a university setting. *Results*: Feedback from patients highlighted the need for an inclusive, evidence-based intervention that would benefit patients experiencing all levels of pain, with differing motivations for recovery. Patients desired social support with others who have had similar surgery to reduce the burden of isolation during rehabilitation. Furthermore, patients valued the involvement of their partners and carers in their rehabilitation, to provide social support and guidance on recovery. Patients also expressed a need for consistent information and expert guidance on all aspects of their recovery. *Conclusions*: These findings can be used to guide the design of rehabilitation interventions following hip and knee replacement and ensure that patient perspectives inform future practice.

## 1. Introduction

Total hip and knee replacement surgeries are amongst the most common elective procedures in the United Kingdom (UK) [1]. In England, Wales, and Northern Ireland, 95,677 primary hip replacements and 103,617 primary knee replacements were recorded in the National Joint Registry in 2019 [1], before the COVID pandemic hit. The UK National Institute for Health and Care Excellence (NICE) guidelines [2] on rehabilitation post-discharge state that hip and knee replacement patients should be advised on self-directed rehabilitation before they leave the hospital, with supervised rehabilitation only offered to patients with functional or cognitive difficulties. In practice, there is much variability in rehabilitation in terms of whether it is supervised or self-directed, what the rehabilitation consists of, where it is delivered, and how long it is provided for [3].

At present, there is no strong evidence to suggest that a single type of exercise-based physical rehabilitation improves recovery for most patients, with research suggesting that home-based self-directed rehabilitation can be as effective as supervised rehabilitation delivered in outpatient clinics [4,5,6]. However, there is little in the literature on how patients experience self-directed rehabilitation. A qualitative study of patient experience following surgery [7] found that some patients did not know how to access services on returning home, and many required additional support in managing issues such as post-discharge pain. Patients wanted a more personalised service, with individualised exercises and goals.

We therefore invited patients who had undergone total hip or knee replacement surgery to take part in one of two advisory groups so that we could understand their views on their rehabilitation needs, how they felt these had been met by the service they received, and what kind of service they would like to have been offered. Patient involvement is recognised as playing an essential part within the UK National Health Service (NHS) in ensuring that services delivered meet patients’ needs and inform staff education and healthcare policies [8,9]. Patient and public involvement (PPI) has been defined by INVOLVE (part of the National Institute for Health Research (NIHR)) as a process “conducted with, or by members of the public, rather than to, about, or for them” [10].

## 2. Methods

This paper uses the INVOLVE definition of PPI and is reported using the Guidance for Reporting Involvement of Patients and the Public (GRIPP 2) [11]. Patients who had undergone hip or knee replacement surgery were invited to one of two advisory group sessions and asked about their rehabilitation following surgery, so that their views could be used to inform future service provision and research. Both groups were co-facilitated by the same two facilitators, a senior physiotherapist (TW) and a health scientist (JG). Prior to commencing each advisory group, the facilitators explained the purpose of the advisory group and sought permission to audio-record the sessions. 

## 3. Ethical Considerations

Ethical approvals were not required for this project, as it involved the public in the planning and design stage of service development and research [12]. Nonetheless, the principles of research governance were still applied, with all information kept confidential. Participants were aware of their right to withdraw at any time.

## 4. Recruitment

Between April and May 2017, an advertisement flyer was circulated via a University’s ‘Public Involvement in Education and Research Partnership (PIER)’. The flyer invited people who had undergone hip or knee replacement surgery to attend a patient advisory group to inform the design of an intervention to facilitate self-management of rehabilitation following surgery. Patients and the public who registered an interest with PIER were contacted by telephone or email to see if they could participate in one of two groups. The two patient advisory groups took place in a meeting room at a UK university in the daytime. Refreshments were provided. 

## 5. Participants

Twenty-one patients registered with PIER their interest in taking part in the groups, and two patient engagement advisory groups were organised. The first (*n* = 9) comprised two men and seven women, with five total hip replacements and four total knee replacements within the last two years (range 4–25 months). The second group (*n* = 5) comprised three women and two men who had had hip replacement surgery between three months and seven years before the group discussion. Ages ranged from 56 to 75 years old. The groups included residents of Dorset and Hampshire who had received treatment from the National Health Service (NHS) or been treated privately in the UK.

### Data Collection and Reporting

The facilitators developed a guidance sheet of questions with prompts to keep the conversation relevant to the group’s purpose. The questions raised in each advisory group were:

- What was particularly challenging trying to recover after discharge?
- What do you think were the most important factors for your rehabilitation?
- How could these factors be utilised in an intervention?

Immediately after each advisory group, the facilitators discussed and noted the main points raised in the group discussions (JG, TW). Then, transcripts were typed up separately for the two groups (with two weeks between groups), with annotations added to the respective transcripts by one facilitator (JG). Two researchers (TI, LB) then independently analysed the data for critical themes. Any discrepancies between findings were resolved and refined through discussion with the research team (JG, TW, TI, LB).

## 6. Results

Information provided by the participants during group discussions has been summarised in Table 1, along with the impact these comments may have on the design of a rehabilitation intervention after hip or knee replacement surgery. The first group comprised former hip and knee replacement patients, and discussions in the group focused mainly on the challenges of rehabilitation and important factors in their recovery. The second group comprised former hip replacement patients only, and although they started with discussions on challenges and important factors in their recovery, they progressed to discussing how they would like a rehabilitation intervention structured. 

In discussions, both groups highlighted how experiences varied; everyone had unique circumstances. One person said:

*“I think the pain in the scar was my problem. The pain was so bad …, it wasn’t actually in the hip. It was in the scar, I think it was muscle damage, so it made it very difficult doing virtually everything.”*

A second commented:

*“I had a real problem with the tourniquet bruising on my thigh, which prevented me from doing a lot of the exercises straight away.”*

However, another piece of feedback was:

*“I did all the exercises they gave me when I left the hospital. You know, walking up the stairs so many times. I did all that in the required amount of time.*

*When it came to an end, I forced myself to go out walking every afternoon, as I like walking anyway.*

*Down the beach for two or three miles, rain or shine. I had no pain in my hip either during or after the operation or ever since. I have had no pain whatsoever.”*

Participants reported a wide range of motivations to recover, such as the need to return to work; the ability to care for family dependents; and the ability to return to leisure and physical activities, such as walking, driving, dancing, and yoga. A desire to return to ‘normal life’ was described by participants. Furthermore, both groups expressed frustration in completing activities of daily living (such as getting in and out of the car and the bath) in the postoperative phase. One participant highlighted that their motivation for recovery differed from that of their younger counterparts, suggesting any rehabilitation intervention would need to be inclusive and tailored to benefit patients of all ages with differing motivations and in varying situations. They said:

*“I am mid-eighties. Recovery motivation is quite different than if you are forties.”*

Another spoke of their family commitments:

*“Being able to care for my father, as I am an only child, and being able to help my younger daughter, as she is a single parent with a young family who has to work to support herself. That was my motivation.”*

Both groups agreed that advice provided within the resources available before surgery and on recovery post-surgery could have been more consistent. They reported that although most people were provided with some exercises following surgery, there was scant information on the specific exercise dose, goals for rehabilitation, and when and how to progress exercises. Some were worried about progressing too quickly and causing irreversible damage, whereas others felt they came off their crutches too early. Participants reported trying to find more advanced exercises once they were ready to progress but that it was easier to know which to choose with professional or specialist guidance. One participant said:

*“You get the initial exercises, but it is very hard to find the next set of exercises. I have gone on the internet and looked for them, and things like that, but obviously my physios help me, but there are lots out there.”*

One lady highlighted the common concerns of self-pacing, lack of progression, and an inability to self-assess:

“*My biggest worry was, am I going too fast? … What I had been given fairly soon became boring, and it wasn’t enough to challenge me. I didn’t know when it was OK to go without crutches. I sent off for a walking stick but didn’t know if it was okay to go with that.*”

Another reported concerns on exercise progression:

*“They don’t tell you how long to carry on for. I thought once I am walking okay, I don’t need to do the exercises, but when I stopped exercising, I started going backwards.”*

Another reported a loss of motivation upon returning to the hospital for a follow-up appointment:

*“I came home with comprehensive instructions, which I didn’t find difficult, and I obviously progressed. When I went back to the hospital and saw the physio, she looked in horror and went, “I don’t want you doing any more like that”. I felt completely deflated as I thought I had been doing really well”.*

The groups discussed the problems of feeling isolated before and following the surgery. They discussed difficulties related to sleep quality due to pain and subsequent fatigue and how their fatigue affected recovery. One member commented:

*“The mental aspect of this whole thing, of being isolated and unwell. It is really bad for your brain; you get extremely depressed if it has been going on for a long time before it is sorted out”.*

Another member spoke about her elderly aunt who had had a hip replacement:

*“She was fit before she had the operation, and the operation was a success. The six weeks of being in took her confidence away and she never went out again”.*

A third suggested that meeting in a group may help to relieve feelings of isolation:

*“It would be really nice to meet up with other people, sit down and have a chat. I do not necessarily do exercises, but I think that is the time when it can be very isolating [in the 6 weeks following surgery]. You are stuck at home; you can’t do your usual life”.*

When asked what they felt were the essential factors in their rehabilitation, the groups talked about the importance of being able to contact a qualified health professional, ideally on a one-to-one basis. They also highlighted the need for evidence-based resources that provide detailed information on recovery following surgery and whom to contact for further information, mainly if they had a problem.

The groups discussed the importance of managing their expectations and setting achievable goals in their rehabilitation. They expressed a desire for their partners and/or carers to be included in discussions regarding their recovery, as it was vital that they also had their expectations managed by someone they trusted and would come to rely upon in long-term recovery.

One woman reported that her partner was over-cautious:

*“My husband thought after a week I would be able to do all the cooking, but he still said “careful” every time I went down a step”.*

Another patient advisor said:

*“My wife thinks she is a doctor as well. She held me back a lot, whereas I think if she had spoken to some other people who had perhaps progressed faster, I would have done too”.*

Those who had surgery at their local NHS hospital highly valued the 4-week follow-up phone call after their surgery. This telephone call’s purpose was to monitor and review their progress and that of wound healing. This telephone call was found to be particularly helpful, as the nurse was able to answer any questions they had.

## 7. What Could a Rehabilitation Intervention Look Like?

It was agreed that although any suitable rehabilitation intervention would need to be inclusive, it should also be able to personalise treatment depending on patients’ circumstances. A participant commented:

*“I don’t think anybody really sits you down and says right, where do you live, what do you do, what is available to you within a reasonable distance so you can access it.”*

The benefits of physical activity, including strengthening exercises, walking, gardening, aqua aerobics and cycling, and body weight management were discussed. When asked which strength exercises rehabilitation professionals prescribed them, answers included stair climbing, cycling, aqua aerobics, pushing against the wall, leg exercises, planks, and dynamic exercises with resistance bands.

One group member suggested holding exercise classes like the ones she was still attending as part of her cardiac rehabilitation. In this intervention, patients are offered components of health education, advice on risk reduction, graded physical activity and lifestyle management advice [13]. The intervention is delivered continuously in a community setting (such as a leisure centre), and patients may attend for as long as required. The cost of the programme (around USD 7.00 per class) was deemed affordable by the group and considered reasonable in terms of quality and cost.

It was agreed that this type of group format would be suitable, with about 20 min for an education component followed by time for questions to be asked. This would be followed by supervised exercise activities such as strengthening exercises. The classes could take place in a community setting, such as a church hall, which should be easily accessible with parking and toilets.

The group was eager to include spouses and carers in the classes so that they could receive additional insights into the recovery process, as well as have an opportunity to experience the exercises themselves. It was also thought that if patients and spouses or carers took part in the classes, they would motivate each other to progress, creating a community network and a level of empathy and understanding. The facilitators asked whether it would be beneficial to include an option for refreshments and time to socialise with others at the end of each session. This ‘social time’ would allow attendees to socialise and discuss their experiences with others undergoing the same journey. Participants agreed that this would be a good idea, particularly for those at higher risk of isolation, that is, those lacking family and social support networks.

Some participants suggested that they would be ready to start rehabilitation six weeks following surgery. Others, however, discussed that the first six weeks after the operation were the hardest due to social isolation, difficulty sleeping, and limited ability to complete activities of daily living. It was agreed that the phone call from nurses or physiotherapists at 3–4 weeks post-surgery for those on the NHS pathway was very beneficial as, at this stage, some were unsure as to how to progress their specific exercises or whether they should still be taking precautions following their operation.

The second group explored who should facilitate exercise rehabilitation sessions. They felt that it should be someone with expert knowledge of rehabilitation following joint replacement surgery, such as a physiotherapist, who could provide individual and group advice and coach the group. The group felt it was important that an exercise specialist individually assessed people before taking part in the classes so that exercise prescriptions could be tailored to the individual.

There was some discussion as to whether mobile apps or smartphones could be used to support the intervention. However, some participants expressed concern about the technology involved as they had not previously used the internet. The second group was asked whether they would be interested in listening to an online podcast which provided guidance on self-managing recovery. Although this was attractive to some, two advisors were wary regarding the complexity of using the internet. A more appealing online media appeared to be an online forum, as group members were keen to access professional feedback in the weeks following surgery.

## 8. Discussion

Our findings highlight the need for inclusive, evidence-based rehabilitation pathways that would benefit patients experiencing all levels of pain, with differing motivations for improving. Patients desired contact with others who have had similar surgery and the involvement of their partners and carers in their rehabilitation. Preliminary work found that patient information leaflets for hip and knee replacement surgery do not provide consistent, evidence-based information and lack advice on personalised exercise prescription [14]. Similarly, participants in the present study expressed a need for consistent information and expert guidance on all aspects of their recovery upon hospital discharge. Participants suggested developing a community-based group exercise intervention, delivered in the weeks following surgery, to help provide social support and expert guidance on rehabilitation, which may help to promote effective, long-term self-management of their recovery.

These findings are consistent with previous investigations of patient experience of hip and knee replacement on an enhanced recovery pathway [7,15,16,17,18,19,20]. In these studies, patients expressed the need for more individualised exercise instructions [7] with a physiotherapist to help them understand when and how to progress their rehabilitation [15,17]. Whilst patients appreciated the short length of stay often associated with enhanced recovery pathways, many felt vulnerable once at home and in need of further support and guidance from professionals [16,18]. Patients also highlighted the importance of their friends and family in their rehabilitation and suggested that they would be unable to manage their recovery without them [19]. In recent years, there has been growing recognition of the vital role of friends and family in patient care, as they play a crucial role in promoting positive health behaviours, empowering patients and assisting with transitions in care [21]. Evidence suggests that those who are socially supported in the postoperative phase may recover quicker and use less pain medication than those with lower social support [22]. Feedback from the participants in this study further highlighted the importance of social support and the involvement of family and friends in the recovery phase after joint replacement surgery.

A key insight from this study is one participant’s observation regarding their cardiac rehabilitation experiences following heart surgery. The cardiac rehabilitation model, one of combined education and graded exercise that may be attended for as long as a patient requires, is perhaps one that could be replicated following joint replacement. The cardiac rehabilitation programme is located within a leisure centre, and the willingness of patients to pay for the session is also significant, highlighting that services may be provided within leisure facilities and within the community rather than the hospital. Indeed, whilst the observations from this study relate to hip and knee replacement surgery, it may be that community-based group rehabilitation interventions may have utility across general surgical populations too, where ERAS principles are implemented, but rehabilitation provision is poorly provided and understood [23]. While the importance of ongoing care to prevent complication or readmission is recognised, the quality of research evidence in postoperative rehabilitation for joint replacement has been rated as low [3]. Incorporating patient feedback into the design of future rehabilitation interventions may improve the quality of the evidence base and ensure that patient perspectives inform practice.

Personalising rehabilitation care plans can be complicated due to limited resources. However, group education and exercise interventions, such as the cardiac rehabilitation programme, are cost-effective when compared to individual physiotherapy sessions. Getting patients back to their normal daily activities such as work or caring for their family, reducing isolation, and giving them confidence in their recovery provide a strong social and financial argument for this type of intervention to be developed and evaluated.

## 9. Limitations

There are several contextual and process factors that may have influenced the findings of this patient and public involvement process. Patient and public involvement commonly attract a certain socio-demographic group motivated by their individual needs and interests in research specific to their condition [24]. While these patients may not represent the general population undergoing hip or knee replacement, their feedback provides an important foundation for understanding this patient population. Although only 14 patients took part in the exercise, this is in line with other PPI work [25,26]. Further consultation is planned with a wider range of participants once future interventions are planned.

The presence of a physiotherapist may have influenced the discussions between patients and the socioeconomic background of the groups (including advisors who were highly educated and/or could afford private healthcare). Patients typically seek advice from their clinicians, and the patient and public involvement model challenges this traditional clinical–patient relationship, where the patient is now required to collaborate, argue, and challenge the clinician [27]. The group environment can benefit discussions as group members can stimulate elaboration and expression. However, the group dynamic may also influence the interaction and response patterns within the group as members listen and respond to the suggestions of others [28]. It should be noted, too, that the length of time since surgery ranged from three months to seven years, so it is likely that memories from seven years ago are less precise than those from three months ago.

## 10. Conclusions

Patients who had undergone hip or knee replacement surgery highlighted the need for an evidence-based intervention to benefit patients experiencing all pain levels, with differing motivations for improving. Patients desired social support with others who have had similar surgery to reduce the burden of isolation during rehabilitation. Furthermore, patients valued the involvement of their partners and carers in their rehabilitation. Patients expressed a need for consistent information and expert guidance on all aspects of their recovery. These findings can guide the design of rehabilitation interventions for patients recovering from hip and knee replacement.

## Figures and Tables

**Table 1:** Feedback from patient advisory groups, and its impact on design of intervention to improve rehabilitation.

Question	Feedback Received	Impact on Design of Intervention
What was particularly challenging during rehabilitation?	The amount of pain experienced following surgery differed between the people in the groups. Some felt very restricted in their activities as their pain was worse than expected, whilst others experienced less pain and were able to complete the exercises given.	Needs to be inclusive and benefit patients experiencing all levels of pain, with differing motivations for improving.
	Motivation varied. Some in the groups were very motivated to improve quickly, “to return to normal” as they had family or work commitments or wanted to continue with activities such as dance and yoga.	
	Inconsistent information was given pre-surgery on resources available to help with rehabilitation, e.g., how to organise home, specialist exercise/swimming classes at local leisure centres.	Information to be available on accessing resources at the hospital and in the community.
	Lack of guidance on how long to continue with the exercise given, at what intensity, how to progress with the exercises and changes in range of motion to be expected—no more advanced exercises given. Some worried about performing exercises too quickly and coming off crutches too early.	Expert guidance required on type and intensity of post-surgery exercise, and how to progress exercises.
	Inconsistent information given on precautions to take after surgery, e.g., how long to wear compression stockings; time before they can drive; how long they have to sleep in a particular position; how to kneel (for total knee replacement surgery). Advice on how to get out of bed; taking laxatives and cut toenails also mixed.	Evidence-based guidance required on precautions required post-surgery, and how long these are necessary, along with advice on managing activities of daily living.
	Too much information online, hard to assess quality.	
	Isolation. Lack of contact with other people. Inability to drive in weeks following surgery.	Contact with others who have had similar surgery, and time to talk together and compare notes/offer support.
What were the most important factors in rehabilitation?	Being able to speak to someone one-on-one.	Ability to speak face-to-face with a specialist health professional.
	Having expectations managed, and the setting of achievable goals.	Guidance from a specialist health professional.
	Having a good booklet giving information and contacts.	Online information/booklet providing current evidence and guidance, and contacts for local services and resources.
	Being advised on how to set up home and assistive equipment available.	Advice required on assistive equipment available for the home.
	Partner/carer having realistic expectations of patient following surgery, so they do not push too hard or hold patient back.	Intervention to include partners and carers so that they have appropriate expectations, and an understanding of the rehabilitation process.
	Other activities undertaken: walking, hydrotherapy, aqua aerobics, static cycling.	Include exercise in the intervention.
	Losing weight (if required).	
	Having a follow-up telephone call with physios/nurses at 3–4 weeks.	Have regular access to a specialist health professional.
How could these factors be utilised in an intervention?	Have classes similar to those given to patients undergoing rehabilitation for cardiac surgery.	
	Have education and exercise components, with Question and Answer session at the end.	
	Hold in a big space accessible to communities such as church halls.	
	Partners take part in group as well to join in with the social aspect and understand the recovery process better.	
	Allow for time for people to socialise with other people in same situation.	
	A follow-up telephone call with physios/nurses should occur at 3–4 weeks post-surgery as per NHS standard practice, and then start intervention at 6 weeks.	
	Have trained facilitator with good knowledge of condition and rehabilitation, who has knowledge of medical history of those attending.	
	Potential for online forum where people could ask questions, or the use of smartphones/apps.	

## Data Availability

Not applicable.
